# Guidewire impaction during percutaneous dilatational tracheostomy

**DOI:** 10.4103/0019-5049.79888

**Published:** 2011

**Authors:** Pramendra Agrawal, Babita Gupta, Nita D’souza, Kapil Dev Soni, Chandni Sinha

**Affiliations:** Department of Anaesthesia and Critical Care, Jai Prakash Narayan Apex Trauma Centre, All India Institute of Medical Sciences, New Delhi, India

**Figure 1:**
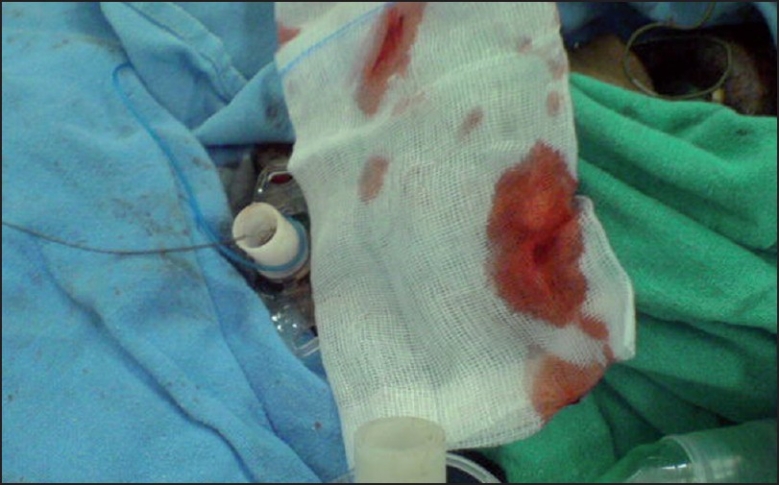
Guidewire coming out through oral route along with endotracheal tube

Sir,

Percutaneous dilatational tracheostomy (PDT) is a frequently carried out procedure in a critical care setting. It is performed in majority of patients by intensivists bedside under endoscopic guidance. Though simple PDT is not devoid of complications, especially if done without endoscopic guidance. We discuss an unusual complication during PDT. A PDT was planned in a 45-year-old male patient with head injury. The patient was placed on a regimen of 1.0 FiO_2_ (Fraction of inspired oxygen). Blood pressure, cardiac rhythm and oxygen saturation were continuously monitored. Analgesia, sedation and neuromuscular blockade were administered. The neck was extended and antiseptic solution on the surgical field was applied. The endotracheal tube was repositioned above the site of the proposed tracheostomy under bronchoscopic guidance. The endotracheal tube cuff was deflated and it was withdrawn to just below the vocal cords. During the insertion of the introducer needle, the lamp of the fibrescope stopped functioning. We decided to proceed without endoscopic guidance since the needle was introduced. Griggs technique was performed using the percutaneous tracheostomy kit. At the end of the procedure, we were unable to pull out the guidewire through the tracheostomy tube. When the endotracheal tube was pulled out, the guidewire also came along with it [Figure 1].

Griggs *et al*.[1] reported the guide wire dilatation forceps (GWDF) technique in 1990. Van Heerden *et al*.,[2] published a series of 54 patients of PDT. They used a bronchoscope for the first 15 cases and found that bleeding and damage to the endotracheal tube were the most common complications. In our case, the introducer needle must have pierced the Murphy’s eye, causing the guidewire to get entangled. Studies with PDT performed with endoscopic guidance[3–6] have reported lower complication rates than studies performed with ‘blind’ PDT. Perioperative and late complication rates for endoscopic and non-endoscopic PDT have been reported to be: 7.2% versus 8.2%, 3.9% versus 6.1% and 1% versus 2.2%, respectively. The mortality rates were 0.65% and 0.52%, respectively, for endoscopically guided and ‘blind’ PDT.[7] Although bronchoscope-guided PDT is advisable, there are still many centres where bronchoscope is not available and blind PDT is done.

This complication re-emphasises the use of bronchoscope during all PDT procedures.
